# A high protein diet (3.4 g/kg/d) combined with a heavy resistance training program improves body composition in healthy trained men and women – a follow-up investigation

**DOI:** 10.1186/s12970-015-0100-0

**Published:** 2015-10-20

**Authors:** Jose Antonio, Anya Ellerbroek, Tobin Silver, Steve Orris, Max Scheiner, Adriana Gonzalez, Corey A Peacock

**Affiliations:** Exercise and Sports Sciences, Nova Southeastern University, 3532 S. University Drive, University Park Plaza Suite 3532, Davie, FL 33314 USA

**Keywords:** Protein, Diet, Body Composition, Nutrition, Body Fat

## Abstract

**Background:**

The consumption of a high protein diet (>4 g/kg/d) in trained men and women who did not alter their exercise program has been previously shown to have no significant effect on body composition. Thus, the purpose of this investigation was to determine if a high protein diet in conjunction with a periodized heavy resistance training program would affect indices of body composition, performance and health.

**Methods:**

Forty-eight healthy resistance-trained men and women completed this study (mean ± SD; Normal Protein group [NP *n* = 17, four female and 13 male]: 24.8 ± 6.9 yr; 174.0 ± 9.5 cm height; 74.7 ± 9.6 kg body weight; 2.4 ± 1.7 yr of training; High Protein group [HP n = 31, seven female and 24 male]: 22.9 ± 3.1 yr; 172.3 ± 7.7 cm; 74.3 ± 12.4 kg; 4.9 ± 4.1 yr of training). Moreover, all subjects participated in a split-routine, periodized heavy resistance-training program. Training and daily diet logs were kept by each subject. Subjects in the NP and HP groups were instructed to consume their baseline (~2 g/kg/d) and >3 g/kg/d of dietary protein, respectively.

**Results:**

Subjects in the NP and HP groups consumed 2.3 and 3.4 g/kg/day of dietary protein during the treatment period. The NP group consumed significantly (*p* < 0.05) more protein during the treatment period compared to their baseline intake. The HP group consumed more (*p* < 0.05) total energy and protein during the treatment period compared to their baseline intake. Furthermore, the HP group consumed significantly more (*p* < 0.05) total calories and protein compared to the NP group. There were significant time by group (*p* ≤ 0.05) changes in body weight (change: +1.3 ± 1.3 kg NP, −0.1 ± 2.5 HP), fat mass (change: −0.3 ± 2.2 kg NP, −1.7 ± 2.3 HP), and % body fat (change: −0.7 ± 2.8 NP, −2.4 ± 2.9 HP). The NP group gained significantly more body weight than the HP group; however, the HP group experienced a greater decrease in fat mass and % body fat. There was a significant time effect for FFM; however, there was a non-significant time by group effect for FFM (change: +1.5 ± 1.8 NP, +1.5 ± 2.2 HP). Furthermore, a significant time effect (*p* ≤ 0.05) was seen in both groups vis a vis improvements in maximal strength (i.e., 1-RM squat and bench) vertical jump and pull-ups; however, there were no significant time by group effects (*p* ≥ 0.05) for all exercise performance measures. Additionally, there were no changes in any of the blood parameters (i.e., basic metabolic panel).

**Conclusion:**

Consuming a high protein diet (3.4 g/kg/d) in conjunction with a heavy resistance-training program may confer benefits with regards to body composition. Furthermore, there is no evidence that consuming a high protein diet has any deleterious effects.

## Background

The protein requirements of active individuals are the subject of much debate. According to the International Society of Sports Nutrition, their Position Stand on Protein specifically states that “protein intakes of 1.4 – 2.0 g/kg/day for physically active individuals is not only safe, but may improve the training adaptations to exercise training.” [1] Other investigators have suggested a 1.2 – 1.4 g/kg/day and 1.6 – 1.7 g/kg/day for endurance and strength-trained athletes, respectively [2]. Thus, a ceiling of 2.0 g/kg/day is ostensibly the consensus in relation to the protein needs of athletic individuals. Data on high protein diets are somewhat misleading in that investigators operationally define ‘high’ incorrectly. The definitions of a high protein diet include: intakes greater than 15–16 % of total energy, as high as 35 % of total calories or intakes that merely exceed the RDA [3]. One study examined the effects of progressive resistance training combined with a protein-enriched diet (i.e., via consuming more lean red meat) on various indices of body composition, physical performance and health in 100 women aged 60–90 years. The investigators discovered that a protein-enriched diet equivalent to approximately 1.3 g/kg/day achieved through lean red meat was a safe and effective way for enhancing the effects of weight training on lean body mass and muscle strength in elderly women [4]. This aforementioned study would fit the published definition of a high protein diet. Another investigation ascertained the effects of an isoenergetic high-protein diet on strength and fatigue in ten young active women during high-intensity resistance exercise. They followed a control diet (55, 15 and 30 % of energy from carbohydrate, protein and fat, respectively) and a high-protein diet (respective values, 30, 40 and 30) for 7 days each in a random counterbalanced design. Subjects on the isoenergetic high-protein, moderate-fat diet maintained muscular strength and endurance during high-intensity resistance exercise without experiencing fatigue earlier compared with a control diet [5]. That study suffers from two profound drawbacks. First, seven days hardly constitutes a ‘diet.’ And secondly, basing a diet on percentages can be quite misleading. Instead, one should operationally define what a ‘high’ protein diet is via the amount consumed daily per kilogram of body weight. Inasmuch as 2.0 g/kg/d seems to be the upper limit of what active individuals purportedly need, it is our contention that for a diet to truly be considered high in protein, daily consumption should necessarily exceed 2.0 g/kg/d. Previous work from our laboratory examined a true high protein diet (4.4 g/kg/d). In essence, consuming over five times the recommended daily allowance of protein had no effect on body composition in resistance-trained individuals who otherwise maintain the same training regimen. That investigation was the first interventional study to demonstrate that consuming a hypercaloric, high protein diet does not result in any significant body composition alterations [6]. Thus, the purpose of the present investigation was to determine the effects of a high protein diet (>3 g/kg/d) combined with a traditional heavy resistance training program on indices of body composition, performance and health in resistance-trained individuals.

## Methods

### Participants

Seventy-three resistance-trained subjects volunteered for this investigation. Subjects were unequally randomized to a control (normal protein intake or NP) and a high protein (HP) group. The purpose of unequal randomization was to take into account the loss of subjects from potential lack of compliance due to the high protein diet as well as gaining additional information on the treatment itself. [7] Participants were otherwise healthy resistance-trained men and women who had been weight training regularly. Individuals in the NP group were instructed to maintain the same dietary habits over the course of the study. On the other hand, subjects in the HP group were instructed to consume ≥3 g protein/kg/d. The extra protein could be obtained from whole food or protein powder. All procedures involving human subjects were approved by Nova Southeastern University’s Human Subjects Institutional Review Board in accordance with the Helsinki Declaration and written informed consent was obtained prior to participation.

### Food diary

Subjects kept a daily diary of their food intake via a smartphone app (MyFitnessPal®). The use of mobile apps for self-monitoring of diets has been previously used [8]. Virtually every subject had previously used this mobile app. Thus familiarity was not an issue except for a small minority. These individuals were taught by the investigators how to properly input data into the app. Note that the MyFitnessPal® app is a database comprised of over 5 million foods that have been provided by users via entering data manually or by scanning the bar code on packaged goods. Thus, the data themselves are primarily derived from food labels (i.e., Nutrition Facts Panel) derived from the USDA National Nutrient database. Thus, in order for subjects to consume a high protein diet, whey or beef protein powder was provided at no cost to the research subjects. However, they were not required to consume protein powder. The rest of their dietary protein was obtained from their regular food intake.

### Body composition

Height was measured using standard anthropometry and total body weight was measured using a calibrated scale. Body composition was assessed by whole body densitometry using air displacement via the Bod Pod® (COSMED USA, Concord CA). All testing was performed in accordance with the manufacturer's instructions. Subjects were instructed to come into the lab after a 3-h fast and no prior exercise 24-h prior. They voided prior to testing. Subjects were tested while wearing only tight fitting clothing (swimsuit or undergarments) and an acrylic swim cap. The subjects wore the same clothing for all testing. Thoracic gas volume was estimated for all subjects using a predictive equation integral to the Bod Pod^®^ software. Each subject was tested at least twice. The calculated value for body density used the Siri equation to estimate body composition. Data from the Bod Pod® included body weight, % body fat, fat free mass and fat mass. All testing was done with each subject at approximately the same time of day pre and post. The Bod Pod was calibrated the morning of the testing session as well as between each subject.

### Performance testing

Performance testing included the 1-RM for the squat and bench press. Also, maximal vertical jump height, broad jump length and total pull ups (1 set) were assessed. Performance tests were conducted by the university’s strength and conditioning coaches and followed the NSCA’s guide to tests and assessments [9]. All subjects were familiar with the performance tests prior to entering the laboratory. In general, each subject performed the following warm up: 5-min run/bike on a treadmill or cycle ergometer at a self-directed easy pace. Subsequently, the performed a dynamic war-up that consisted of 10 yards each of high knees, butt kicks, side shuffles, and karaoke. This was followed by 10 push ups and 10 body weight squats. Subjects then rested for 2–3 min prior to commencing the performance tests. Subsequently, the following tests were performed in the order given: vertical jump – highest value with a maximum number of three attempts; broad jump – highest value with a maximum number of three attempts. For both the vertical and broad jump, there was a rest interval of approximately 60–180 seconds. This was followed by the bench press and back squat (to parallel). Each subject was allowed one warm-up set followed by a maximum of three attempts. The rest interval between attempts was 120–180 seconds. The final test was the pull-up. Each subject was given one attempt to perform the maximal number of pull-ups. The strength and conditioning coaches who conducted the tests were all certified strength and conditioning specialists (CSCS).

### Blood analysis – basic metabolic panel

A smaller subset of subjects volunteered to have their blood analyzed. They presented in a fasted state at a local Quest Diagnostics™ facility at the same time of day pre and post (i.e., if they came to the clinic in the early morning for pre-testing, they did the same thing post-test). A basic metabolic panel was performed: Calcium, Carbon Dioxide, Chloride, Creatinine with GFR Estimated, Glucose, Potassium, Sodium, Urea Nitrogen (BUN) and BUN/Creatinine Ratio (calculated). Quest Diagnostics performed each test according to the standard operating procedure of the company.

### Training program

Each subject was instructed to follow a heavy resistance program as outlined below. The program was designed to increase strength and lean body mass. Training frequency was five days per week for the 8-week treatment period. The program was a ‘split routine’ in which different body parts were trained on consecutive days as seen in Table 1 and Table 2. Because each subject was already resistance-trained, they were familiar with the exercises provided in the program.

**Table 1:** Training program

Week 1	Week 6
Mon – Chest, Shoulder, Triceps (3 × 15, sets × reps; does not include 2 warm up sets)	Mon – Back, Biceps (3 × 5)
Tues – Hips, Legs (3 × 15)	Tues – Chest, Shoulder, Triceps (3 × 5)
Wed – Back, Biceps (3 × 15)	Wed – Hips, Legs (3 × 12)
Thurs – Chest, Shoulders, Triceps (3 × 10)	Thurs – Back, Biceps (3 × 12)
Fri – Hips, Legs (3 × 10)	Fri – Chest, Shoulder, Triceps (3 × 12)
Sat – REST	Sat – REST
Sun - REST	Sun – REST
Week 2	Week 7
Mon – Back, Biceps (3 × 10)	Mon – Hips, Legs (3 × 8)
Tues – Chest, Shoulder, Triceps (3 × 5)	Tues – Back, Biceps (3 × 8)
Wed – Hips, Legs (3 × 5)	Wed – Chest, Shoulders, Triceps (3 × 8)
Thurs – Back, Biceps (3 × 5)	Thurs – Hips, Legs (3 × 8)
Fri – Chest, Shoulder, Triceps (3 × 12)	Week 8 (Tapering week)
Sat – REST	Mon – Chest, Shoulders, Triceps (3 × 5–15)
Sun – REST	Tues – REST
Week 3	Wed – Back, Biceps (3 × 5–15)
Mon – Hips, Legs (3 × 12)	Thurs –REST
Tues – Back, Biceps (3 × 12)	Fri – Hip, Legs (3 × 15–15)
Wed – Chest, Shoulders, Triceps (3 × 10)	Sat – REST
Thurs – Hips, Legs (3 × 10)	Sun – REST
Fri – Back, Biceps (3 × 10)	
Sat – REST	
Sun – REST	
Week 4	
Mon – Chest, Shoulders, Triceps (3 × 5)	
Tues – Hips, Legs (3 × 5)	
Wed – Back, Biceps (3 × 5)	
Thurs – Chest, Shoulder, Triceps (3 × 12)	
Fri – Hip, Legs (3 × 12)	
Sat – REST	
Sun – REST	
Week 5	
Mon – Chest, Shoulder, Triceps (3 × 12)	
Tues – Hips, Legs (3 × 8)	
Wed – Back, Biceps (3 × 8)	
Thurs – Chest, Shoulders, Triceps (3 × 8)	
Fri – Hips, Legs (3 × 5)	
Sat – REST	
Sun - REST

**Table 2:** Choice of exercises for each body part

Exercise choices	
Chest	(subject performed 3 of these) flat bench press, incline bench press, cable cross-overs, pec deck, flat bench flies, decline bench press
Shoulders	(subject performed 3 of these) – upright row, machine military press, dumbbell overhead presses, lateral dumbbell raises, shoulder shrugs
Triceps	(subject performed 2 of these) – triceps pushdowns, dips, French press
Back	(subject performed 4 of these) – Wide grip lat pulldown, narrow grip lat pulldown, chin ups, cable rows, dumbbell rows, dumbbell flies
Biceps	(subject performed 3 of these) – standing barbell curls, standing EZ bar curl, concentration curls, preacher curls, hammer curls
Legs	(subject performed 5 of these) – Back squats, Smith machine squats, Leg Press, Lunges, Leg curls, Leg extensions, calf raise (seated or standing), Stiff-legged deadlift

The investigators and research assistants were in contact with each subject on a weekly basis to ensure compliance with the exercise training program. Compliance was determined via measurements of volume load (repetitions × sets × weight) which should have increased over the course of the treatment period. Furthermore, subjects were instructed to not perform any aerobic exercise during the treatment period.

### Statistics

A two-time point (Pre, Post) by two-group (NP, HP) repeated-measures analysis of variance (ANOVA) was utilized to analyze the data with a *p* <0.05 considered significant. Post hoc analyses of any significant main effects of condition were performed utilizing t tests with Benjamini and Hochberg false discovery rate correction for multiple comparisons. Data are presented as the mean ± SD. All statistical analyses were completed using SPSS for Windows (version 20.0, SPSS Inc., Evanston, IL).

## Results

Of the 73 participants that were originally enrolled, 48 completed the study and were included in the final analysis. Fifteen and 10 subjects dropped out of the HP and NP groups respectively. Of the 25 who dropped out, 22 did not provide a reason. However, two dropped out due to a non-specific injury while training (one each from the HP and NP groups) whereas one dropped out from the HP group due to military service. Baseline physical characteristics are presented in Table 3. There was a significant difference (*p* < 0.05) between the NP and HP groups regarding years of training experience. Otherwise, no differences existed at baseline.

**Table 3:** Subject characteristics

	Age years	Height cm	Weight kg	Years training
Normal Protein (NP)	24.8 ± 6.9	174.0 ± 9.5	74.7 ± 15.3	2.4 ± 1.7*
*n* = 17 (4 female, 13 male)				
High Protein (HP)	22.9 ± 3.1	172.3 ± 7.7	74.3 ± 12.4	4.9 ± 4.1
*n* = 31 (7 female, 24 male)				

Data are mean ± SD. Legend: *cm* centimeters, *kg* kilograms. *P <0.05 – significant between group differences

### Body composition

**Fig. 1:**
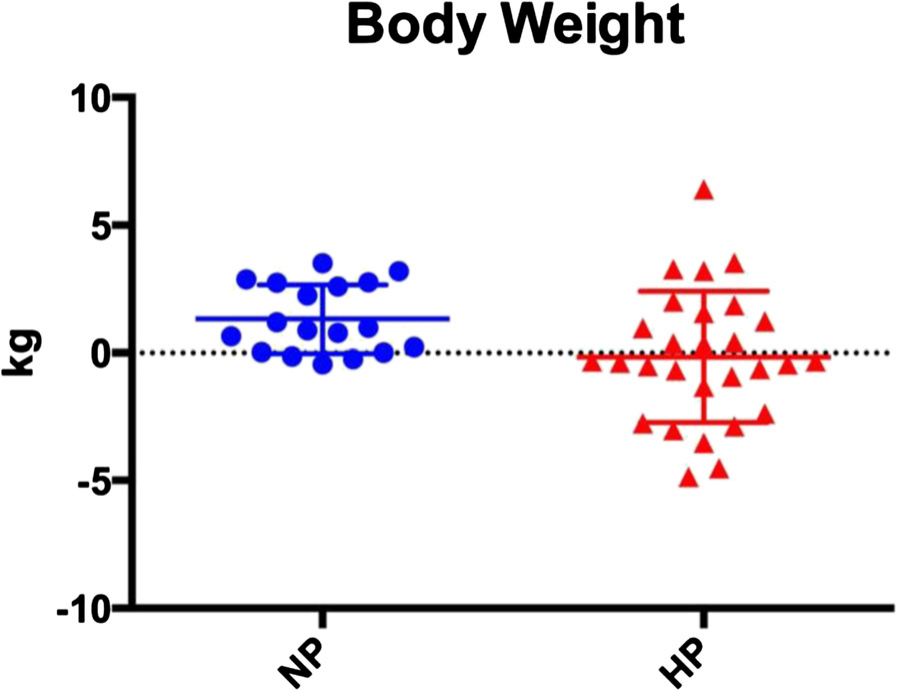
Each data point represents the change for an individual. The horizontal lines represent the mean ± SD. Legend: HP – high protein; NP – normal protein

**Fig. 2:**
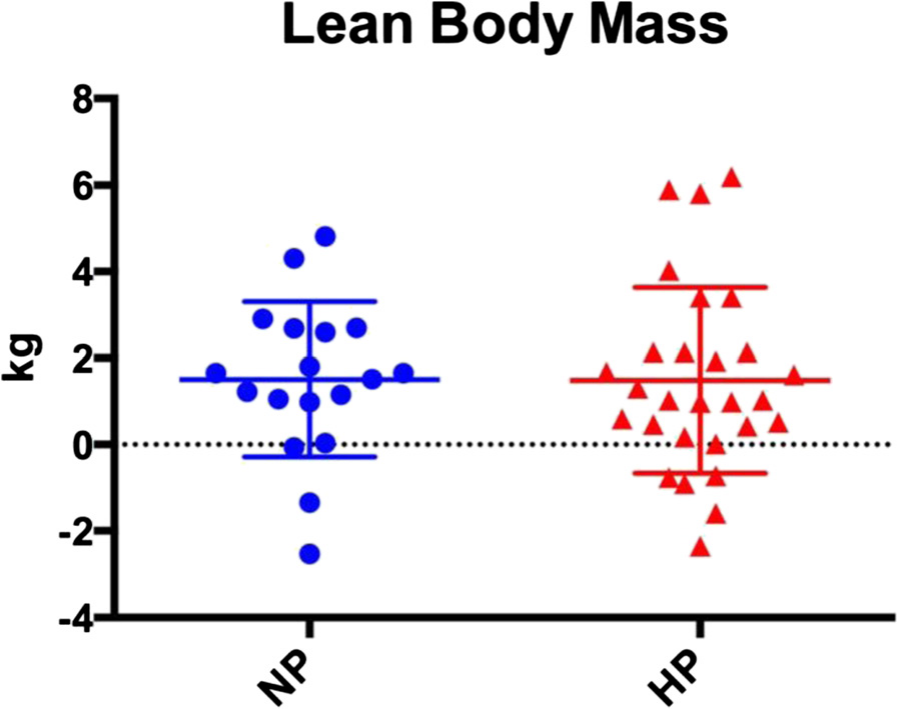
Each data point represents the change for an individual. The horizontal lines represent the mean ± SD. Legend: HP – high protein; NP – normal protein

**Fig. 3:**
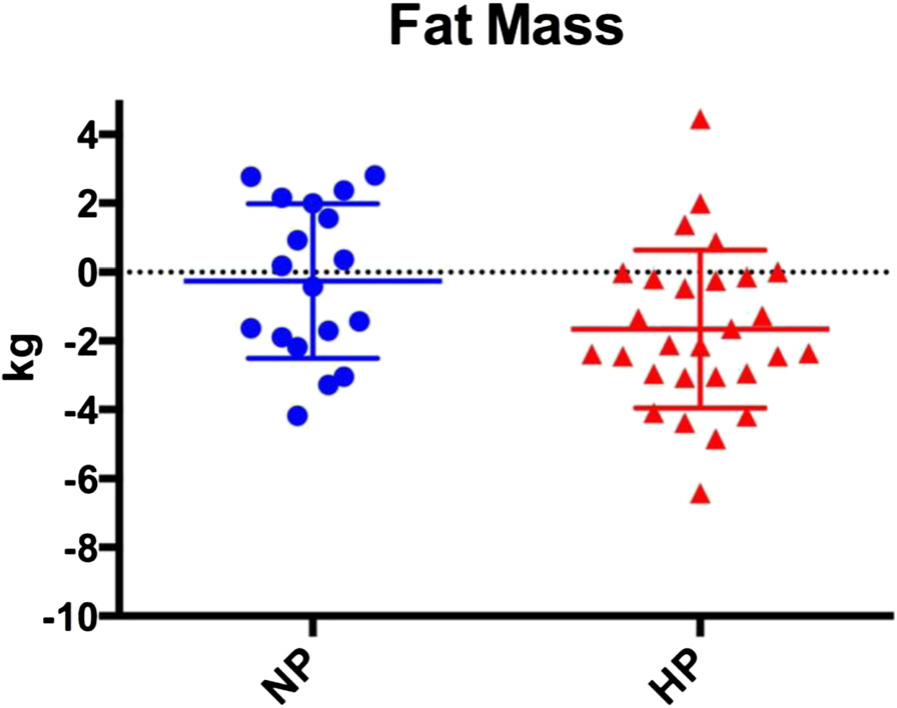
Each data point represents the change for an individual. The horizontal lines represent the mean ± SD. Legend: HP – high protein; NP – normal protein

**Fig. 4:**
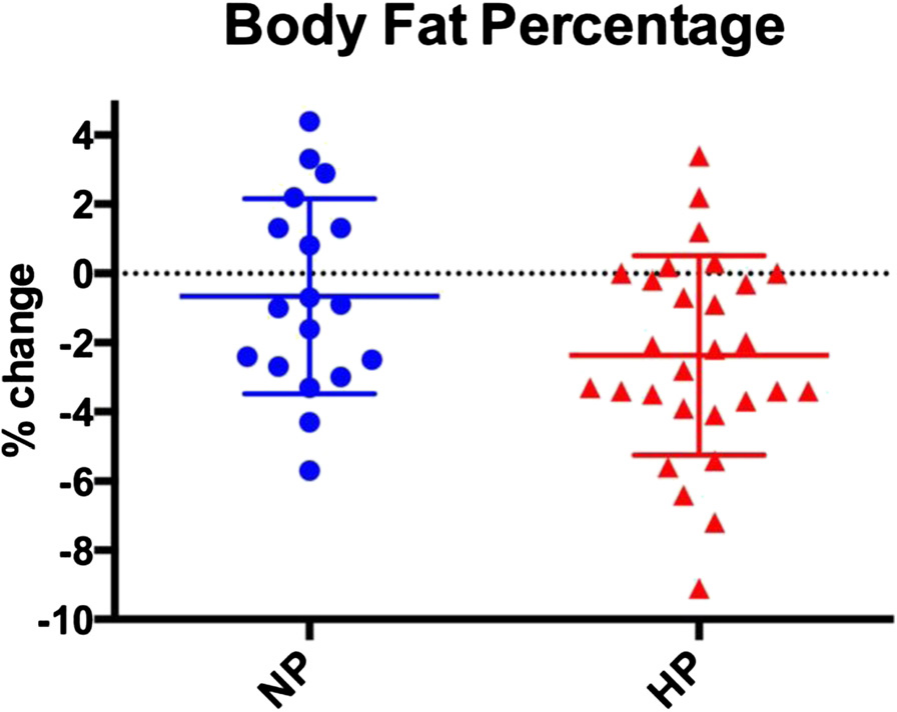
Each data point represents the change for an individual. The horizontal lines represent the mean ± SD. Legend: HP – high protein; NP – normal protein

Body composition changes are presented in Table 4 and Figs 1, 2, 3 and 4. There were no differences between the NP and HP groups for any of the body composition, performance or health variables at baseline. The NP and HP group experienced a significant change (pre vs post) for fat free mass, fat mass and % body fat (*p* < 0.05) (Table 4 and Figs 2, 3 and 4). The NP group also experienced a significant increase in body weight (*p* < 0.05) (Table 4 and Fig. 1). However, between group differences were found for fat mass and percent body fat; the HP group lost an average of 1.6 kg of fat mass versus 0.3 kg in the NP group. Moreover, the percent body fat decrease was −2.4 % and −0.6 % in the HP and NP groups respectively. (Table 4 and Figs 3, 4).

**Table 4:** Body composition

	NP			HP	
	Pre	Post	Pre	Post	Between group
BW (kg)	74.7 ± 15.3	76.0 ± 14.9*	75.8 ± 11.3	75.7 ± 11.9	*P* = 0.04#
FFM (kg)	59.6 ± 13.4	61.1 ± 13.5*	61.4 ± 11.8	62.9 ± 11.3*	NS
FM (kg)	15.1 ± 6.0	14.8 ± 5.4*	13.5 ± 5.6	11.9 ± 5.9*	*P* = 0.04#
% BF	20.2 ± 7.6	19.6 ± 6.8*	18.3 ± 7.7	15.9 ± 7.3*	*P* = 0.05#

Data are mean ± SD. *Denotes significant time effects (Pre vs. Post) (*P* < 0.05). #Denotes significant time by group effects (HP versus NP)

Legend: *BW* body weight, *FFM* fat free mass, *FM* fat mass, *NS* not significant, % *BF* percentage body fat, *HP* high protein, *NP* normal protein

### Exercise performance

The heavy resistance training program produced a significant (*p* < 0.05) increase in 1-RM strength for both the squat and bench press as well as the vertical jump (peak height) and pull ups (maximal number of repetitions) for the NP and HP groups. There were no significant changes in the broad jump for either group (Table 5). Seventy-eight and 93 % of the NP and HP group subjects, respectively, were compliant with the training program (i.e., they showed an increase in volume load over the course of the treatment period).

**Table 5:** Exercise performance

	NP			HP	
	Pre	Post	Pre	Post	Between group
BP (kg)	71.8 ± 32.9	81.4 ± 33.9*	87.8 ± 37.8	94.1 ± 37.9*	NS
SQ (kg)	104.4 ± 48.7	115.8 ± 44.7*	112.0 ± 37.3	120.5 ± 33.1*	NS
VJ (cm)	60.1 ± 11.6	64.5 ± 11.3*	60.9 ± 13.9	64.1 ± 13.6*	NS
BJ (cm)	202.4 ± 38.5	212.7 ± 37.1	218.9 ± 40.2	216.9 ± 39.0	NS
Pull-ups	10.7 ± 8.2	13.6 ± 9.3*	10.7 ± 6.7	14.2 ± 7.8*	NS

Data are mean ± SD. *Denotes significant time effects (Pre vs. Post) (*P* < 0.05). There was no significant time by group effects. Legend: *BP* bench press, *BJ* broad jump, *NS* not significant, *SQ* squat, *VJ* vertical jump

### Diet

There were no significant differences in dietary intake at baseline between the NP and HP groups. The NP group however increased their protein consumption over the treatment period (*p* < 0.05) (total protein and per kg body weight). The HP group consumed significantly more calories and more protein (*p* < 0.05) over the treatment period (total protein and per kg body weight). However, there were between group differences (*p* < 0.05) for dietary energy and protein intake (HP > NP) during the treatment period (Table 6). There were no significant changes in carbohydrate or fat intake in either group nor was there a change in caloric intake in the NP group.

**Table 6:** Dietary intake

	NP		HP	
	Pre	Post	Pre	Post
Kcal	2016 ± 56	2119 ± 57	2240 ± 74	2614 ± 80*#
CHO g	205 ± 76	196 ± 65	223 ± 88	234 ± 11
PRO g	130 ± 48	167 ± 51*	154 ± 49	255 ± 53*#
Fat g	75 ± 20	74 ± 25	77 ± 30	81 ± 33
	Pre	Post	Pre	Post
Kcal/kg/d	28.0 ± 7.6	29.3 ± 7.0	29.3 ± 9.2	35.7 ± 10.7*#
CHO g/kg/d	2.9 ± 1.1	2.7 ± 0.9	3.0 ± 1.3	3.1 ± 1.5
PRO g/kg/d	1.8 ± 0.6	2.3 ± 0.6*	2.1 ± 0.7	3.4 ± 0.6*#
Fat g/kg/d	1.0 ± 0.3	1.0 ± 0.3	1.0 ± 0.4	1.1 ± 0.5
	Pre	Post	Pre	Post
CHO %	40 ± 7	37 ± 6	40 ± 7	34 ± 7*
PRO %	26 ± 9	32 ± 7	28 ± 8	39 ± 9*
Fat %	34 ± 6	31 ± 5	32 ± 6	27 ± 6

Data are mean ± SD

*CHO* carbohydrate, *PRO* protein, *g* grams, *kg* kilograms, *d* days, *HP* high protein, *NP* normal protein. *P < 0.05 – denotes significant increase pre vs post. #*P* < 0.05 – denotes HP post > NP post

**Table 7:** Basic metabolic panel

	NP (*n*=9)		HP (*n*=14)		
	Pre	Post	Pre	Post	Normal values
Glucose (mg/dl)	83.7±11.6	77.4±12.4	84.0±10.3	85.4±11.1	65-99
BUN (mg/dl)	15.7±2.9	17.6±4.7	19.6±5.5	20.7±4.4	7.0-25.00
Creatinine (mg/dl)	1.0±0.2	1.0±0.2	1.1±0.2	1.1±0.1	0.6-1.35
GFR	101.8±12.8	100.1±15.5	90.7±13.8	90.2±9.1	§
BUN/Creatinine	16.6±3.9	18.8±7.5	17.7±6.3	18.5±4.2	6.0-22.0
Sodium (mmol/l)	139.2±1.9	139.6±1.9	139.1±1.5	138.8±2.1	135-146
Potassium (mmol/l)	4.1±0.2	4.0±0.2	4.4±0.3	4.3±0.2	3.5-5.3
Chloride (mmol/l)	103.1±1.4	103.7±1.4	102.6±2.3	102.7±2.0	98-110
CO^2^ (mmol/l)	27.4±2.9	26.7±2.2	27.1±2.9	27.1±1.4	19-30
Calcium (mg/dl)	9.6±0.3	9.5±0.3	9.6±0.3	9.6±0.3	8.6-10.3

Data are mean±SD. Legend: *BUN* blood urea nitrogen, *GFR* glomerular filtration rate (§ normal values: ≥60 ml/min/1.73m^2^). There were no changes in either group; all values were within the normal range. *HP* high protein, *NP* normal protein

### Blood analysis

There were no changes in any of the variables measured as part of the basic metabolic panel (i.e., glucose, calcium, sodium, potassium, CO_2_, chloride, blood urea nitrogen and creatinine; Table 7).

## Discussion

This is the second investigation from our laboratory that has examined the effects of a true high protein diet (i.e., > 2 grams per kg body weight daily). Previously published work has shown that the mere addition of extra protein does not lead to substantive changes in body composition (i.e., no statistically significant change in FFM, fat mass or % body fat) in trained individuals who otherwise do not alter their exercise regimen [6]. This is the first investigation in which a high protein diet in conjunction with a periodized heavy resistance training program was performed; moreover, subjects did not perform any aerobic exercise during the treatment period. In brief, our data suggests that consuming protein well above the recommended dietary allowance (RDA) can favorably alter body composition as long as changes are also made in one’s exercise training regimen. This is in contrast with our original pilot study in which subjects consumed five times the RDA for protein (~4.4 g per kg daily) for eight weeks. In that investigation, there were no statistically significant changes in body composition in the high protein diet group. Thus, this follow-up study was undertaken to ascertain if changing the resistance-training regimen in conjunction with a high protein diet could indeed affect the adaptive response.

It should be noted that other investigators have suggested that trained individuals may have a lower requirement for protein due to increased efficiency of use of protein. Accordingly, “several studies have shown that strength training, consistent with the anabolic stimulus for protein synthesis it provides, actually increases the efficiency of use of protein, which reduces dietary protein requirements” [10]. If indeed regular heavy resistance training enhances efficiency, there would be no effect of added protein vis a vis body composition alterations. In other words, the consumption of protein in amounts far above the RDA should have little to no effect on body composition. Our investigation demonstrates that protein intakes that are approximately 60 % greater than even the highest recommended intakes (i.e., 2 grams per kg body weight daily) produce favorable alterations in body composition when combined with a periodized heavy resistance training regimen.

It has been shown that acute alterations in muscle protein synthesis (MPS) have little to no predictive value regarding chronic changes in lean body mass [11]. Thus, studies that have examined acute changes in MPS are likely poor predictors of body composition in general. For instance, there was a fairly recent investigation of six healthy young men that performed an intense bout of leg-based resistance exercise (i.e., 4 sets of 8–10 repetitions to failure of the leg press, knee extension and leg curls) followed by the randomized consumption of drinks containing 0, 5, 10, 20, or 40 g whole egg protein. They found that 20 grams of egg protein maximally stimulated MPS and albumin protein synthesis after resistance exercise. Furthermore, “dietary protein consumed after exercise in excess of the rate at which it can be incorporated into tissue protein stimulates irreversible oxidation” [12]. This study has been cited as the primary basis for a limitation in protein intake per meal. However, as previously mentioned, acute changes in MPS are a poor predictor of actual gains in fat free mass. Furthermore, this response was examined in egg protein; it is not known if a similar response would be found in whey, beef, casein, soy or any other whole protein source. Even if one were to assume the 20 gram per meal is sufficient to maximize the MPS response, that would translate into 60 grams of protein consumed daily (i.e., if one’s primary protein was consumed over three meals: breakfast, lunch, dinner). That amount is substantially less than what was consumed by the normal and high protein groups in the current study. We would posit that in order to establish causality regarding dietary protein consumption and changes in body composition, time course training studies are perhaps the best way to achieve that.

Hydration status is an important variable that has garnered increased attention because of its impact on body composition assessment. Work by Utter et al. has shown that several methods (e.g., air displacement plethysmography [Bod Pod], hydrostatic weighing and skinfolds) showed a significant decrease in FFM from the hydrated to the dehydrated state [13]. We were unable to measure hydration status; however, we did have participants follow identical pre- and post-testing conditions. In this respect, it is possible that hydration status may have been a contributing factor to the variability we are reporting with our body composition data. Nevertheless, a prudent step for future studies is to determine hydration status prior to body composition assessment.

Our study discovered that consuming protein in amounts that are 3–4 times greater than the RDA result in a similar FFM increase for both the normal and high protein groups; however, the high protein group experienced a significantly greater loss of fat mass compared to the normal protein group in spite of the fact that they consumed on average ~400 kcals more per day over the treatment period. One could speculate the gains in FFM in both groups were the result of providing a different training stimulus than what each subject had previously used. Our prior study showed that the mere consumption of prodigiously high amounts of protein (>4 g per kg daily) had no effect on body composition if training was not altered even though there was a trend towards better body composition. In the current investigation, the high protein group demonstrated greater compliance vis a vis the training program than the normal protein group; perhaps this can explain in part why changes in fat mass were substantially greater in that group.

Alternative explanations for the decrease in fat mass in the high protein group include possible changes in resting and sleep energy expenditure. A recent study examined 25 participants who ate approximately 40 % excess energy for 56 days from 5 %, 15 %, or 25 % protein diets. If the extra calories consumed were from protein, both sleep and resting 24-h energy expenditure increased in relation to protein intake. However, this investigation found no relationship between changes in fat mass and changes in energy expenditure [14]. It should be noted that there was not an exercise component to that study.

Another study examined 12 pairs of identical twins that overfed (~840 kcals extra daily) over 100 days [15]. The average gain in fat mass and FFM were 5.4 kg and 2.7 kg. In comparison, our study showed an average loss of fat mass (0.3 kg normal group, 1.6 kg high protein group) and gain in FFM (1.5 kg for both the normal and high protein group). The authors of the identical twin overfeeding study noted that no single variable was a great predictor of body composition changes [15]. The identical twin overfeeding study did not have an exercise component and did not use trained subjects. Thus, its relevance is questionable in athletic populations that purposefully engage in overfeeding.

In a similar study, participants were fed 140 % of energy needs, with 5, 15 or 25 % of energy from protein, for 56 days. They discovered that changes in vector magnitude (VM), a weight-independent measure of activity and activity-related energy expenditure (AEE) were positively correlated with weight gain; however, protein intake had no effect on changes in activity. Thus, “overfeeding produces an increase in physical activity and in energy expended in physical activity after adjusting for changes in body composition, suggesting that increased activity in response to weight gain might be one mechanism to support adaptive thermogenesis [16]”. Again this study did not have an exercise training component.

It is possible that in the current investigation, changes in AEE and perhaps non-exercise activity thermogenesis (NEAT) might account in part for the greater changes in body composition in the high protein diet group [17, 18]. According to Levine et al., NEAT can vary substantially between individuals by as much as 2000 kcals daily [18]. Obese individuals tend to be seated more than lean individuals up to 2.5 h daily. That could represent an additional 350 kcals expended per day. Thus, it is possible that in the individuals in which NEAT increased the most during overfeeding, they were more likely to lose fat mass. And one could speculate that the more advanced training status of the high protein group might lend itself to greater NEAT.

Certainly, one should not discount the role of protein’s thermic effect (i.e., TEF or thermic effect of feeding). Protein has a TEF of approximately 19–23 % in both obese and lean individuals whereas carbohydrate is approximately 12–14 % [19]. In fact, a high protein meal (45 % total kcal) elicits a 30 % greater TEF than an isocaloric low protein meal (15 % total kcal) in active females [20]. It should be noted that the TEF of fat is substantially less in the obese than in lean subjects [19]. The subjects in our study did not alter fat intake; thus, that could not be an explanation for changes in body composition. One could speculate that subjects in the high protein diet group experienced a combination of enhanced TEF, AEE, NEAT and SEE; this might explain in part the decrease in fat mass. Furthermore, the high protein group was more compliant with the exercise training regimen.

The training regimen used in the current study was clearly effective in producing an adaptive response. Both groups experienced a significant increase in muscular strength, power and endurance. It should be emphasized that it is quite difficult for trained subjects to gain FFM. Thus, the fact that on average both the normal and high protein groups gained 1.5 kg of FFM is an important point. The high protein diet group was more ‘trained’ (i.e., years of training experience) than the normal protein group. This may explain in part why the HP group did not gain more FFM than the NP group. Thus, any gain in FFM and strength may be viewed as a possible result of the extra protein consumed. However, the HP group was also more compliant with the training program and that would certainly be another causative factor in promoting FFM gains.

On average, both groups experienced a gain in FFM and a loss of fat mass; nonetheless, our data demonstrate that there is a bit of individual variability in the response. At the high end, there were subjects in the normal and high protein group that gained up to 7 kg of FFM and lost up to 4 kg of fat mass. Conversely, there were subjects who lost FFM or gained fat mass. In general, our data suggest that vast majority of individuals (~70 %) that consume a high protein diet (>2 g per kg daily) do indeed get an improvement in body composition. A study by Hubal et al. showed that among 585 subjects that underwent a unilateral resistance training program of the elbow flexors, several subjects showed no gain in muscle size whereas others experienced a profound increase [21]. This shows indeed that there is a fairly substantive genetic component to the exercise training response; it would also be reasonable that such a component exists with the addition of a dietary treatment (ex., increased protein intake).

This study also found no harmful effects of consuming a high protein diet on renal function. Thus, professionals who work with athletes (i.e., sports nutritionists, sports dietitians, clinical nutritionists, medical doctors, strength coaches, athletic trainers, etc.) should be aware that athletes can consume very high amounts of protein with no harmful effects over a period of several weeks. Whether side effects will occur over longer protein overfeeding periods has not yet been investigated.

## Conclusion

In conclusion, this is the first randomized controlled trial that has examined the effects of consuming a high protein diet in conjunction with a periodized heavy resistance training regimen over the course of several weeks on markers of performance, health and body composition. This study as well as previous work from our lab suggests that gains in body fat are unlikely to occur with protein overfeeding [6]. Furthermore, gains in FFM can indeed occur in trained subjects with no harmful side effects of high protein consumption. This investigation confutes the notion that trained subjects need only 1.5–2.0 grams of protein per kg body weight daily and that intakes above that are superflous. In fact, we would speculate that the minimal daily needs of dietary protein in trained individuals should be approximately 2 g/kg/d.

Limitations of this study include the fact that the NP group exceeded their baseline protein intake. This was contrary to the instructions given to them. Subjects in this group were instructed to maintain their regular protein intake of ≤ 2 grams of protein per kg daily. Nonetheless, this group ended up significantly increasing their protein intake. However, there was an increase in LBM with no overall changes in fat mass. It should be noted that the HP group had significantly more training experience than the NP group. This would likely result in less of an ability to gain LBM over a finite training period.

Dietary self-reports also present problems vis-á-vis its accuracy. However, much of the additional protein consumed by subjects was in the form of protein powder; it would seem reasonable to assume that subjects could provide an accurate dietary recall with such a simple dietary addition. Furthermore, from an entirely pragmatic perspective, the use of dietary recalls (i.e., our investigation examined intake daily) is the most logical option. Unless investigators literally measure every food and beverage consumed by a subject, it is pragmatically impossible to get 100 % accuracy particularly in free-living subjects. Future work should examine very highly trained athletes who undergo cycles of varying protein intake over a period of several months or years. This would at least provide information in terms of whether the highly trained respond more so to a change in training stimulus, diet or a combination of both.
